# Transcriptional Profiling Reveals Brain Region-Specific Gene Networks Regulated in Exercise in a Mouse Model of Parkinson’s Disease

**DOI:** 10.3389/fnagi.2022.891644

**Published:** 2022-06-23

**Authors:** Weifang Tong, Kunshan Zhang, Hongkai Yao, Lixi Li, Yong Hu, Jingxing Zhang, Yunping Song, Qiang Guan, Siguang Li, Yi E. Sun, Lingjing Jin

**Affiliations:** ^1^Department of Neurology, Tongji Hospital, School of Medicine, Neurotoxin Research Center of Key Laboratory of Spine and Spinal Cord Injury Repair and Regeneration of Ministry of Education, Tongji University, Shanghai, China; ^2^Department of Neurology and Neurological Rehabilitation, Shanghai Yangzhi Rehabilitation Hospital, School of Medicine, Tongji University, Shanghai, China; ^3^The Marlene and Paolo Fresco Institute for Parkinson’s and Movement Disorders, Department of Neurology, NYU Langone Health, NYU School of Medicine, New York, NY, United States

**Keywords:** exercise, Parkinson’s disease (PD), RNA-Seq, brain region specific, synapses

## Abstract

**Background:**

Exercise plays an essential role in improving motor symptoms in Parkinson’s disease (PD), but the underlying mechanism in the central nervous system remains unclear.

**Methods:**

Motor ability was observed after 12-week treadmill exercise on a 1-methyl-4-phenyl-1,2,3,6-tetrahydropyridine (MPTP)-induced mouse model of PD. RNA-sequencing on four brain regions (cerebellum, cortex, substantia nigra (SN), and striatum) from control animals, MPTP-induced PD, and MPTP-induced PD model treated with exercise for 12 weeks were performed. Transcriptional networks on the four regions were further identified by an integrative network biology approach.

**Results:**

The 12-week treadmill exercise significantly improved the motor ability of an MPTP-induced mouse model of PD. RNA-seq analysis showed SN and striatum were remarkably different among individual region’s response to exercise in the PD model. Especially, synaptic regulation pathways about axon guidance, synapse assembly, neurogenesis, synaptogenesis, transmitter transport-related pathway, and synaptic regulation genes, including Neurod2, Rtn4rl2, and Cd5, were upregulated in SN and striatum. Lastly, immunofluorescence staining revealed that exercise rescued the loss of TH^+^ synapses in the striatal region in PD mice, which validates the key role of synaptic regulation pathways in exercise-induced protective effects *in vivo*.

**Conclusion:**

SN and striatum are important brain regions in which critical transcriptional changes, such as in synaptic regulation pathways, occur after the exercise intervention on the PD model.

## Introduction

Parkinson’s disease (PD) is a progressive neurodegenerative disorder caused by degeneration of dopaminergic neurons in the substantia nigra (SN) (Samii et al., 2004). Clinical manifestations of patients with PD mainly consist of rest tremor, bradykinesia, rigidity, and loss of postural reflexes (Jankovic, 2008). Levodopa remains the most common medication for alleviating some of the motor deficits in the early stages of the disease (Lane, 2019). However, chronic use of that drug probably induces side effects, such as levodopa-induced dyskinesia (LID) and impulse control disorders (Voon et al., 2017; Donzuso et al., 2020). Moreover, no specific medicine has been developed to reverse the progression of PD permanently. Therefore, clinicians and researchers have paid more attention on physical exercise.

Recent studies revealed that exercise on patients with PD improves their quality of life and physical function, including locomotion, balance, and gait performance (Goodwin et al., 2008; Shah et al., 2016; Mak and Wong-Yu, 2019; van der Kolk et al., 2019). The observed increments in gait speed and stride length after treadmill and robotic exercise for subjects with PD imply the repairment of nigrostriatal system and cerebellum controlling semi-autonomous stereotypic movements (Hirata et al., 2020; Surkont et al., 2021). In addition, the motor cortex was also found involved in the recovery of PD patients after exercise training (Rothwell, 1999; Fisher et al., 2008). Profound changes occurring in those brain regions during physical exercise may contribute to PD rehabilitation, and underlying molecular mechanisms need further studying.

Previous studies on animal models have demonstrated that exercise practice would modulate the function of striatum, probably by augmenting the dopamine content, improving the mitochondrial function and restricting the extracellular glutamate accumulation (Hendricks et al., 2012; Yue et al., 2012; Chen and Li, 2019). Lee et al. (2018) reported that treadmill running could improve motor balance and coordination in PD rats by suppressing the loss of purkinje cells in the cerebellar vermis.

Despite those clinical and animal studies, molecular mechanisms in the central nervous system (CNS) underlying exercise-induced effects have not been fully elucidated. Especially, most work focused on isolated brain regions (Kintz et al., 2017; Myers et al., 2018), and critical molecules in the cortex, cerebellum, SN or striatum were not overall screened. Systematic analysis of molecular pathways as well as interactions of target brains regions involved in exercise-induced PD rehabilitation is required. Here, we speculate physical exercise in patients with PD may improve motor symptoms by modulating critical molecular networks in those 4 brain region and by affecting the circuit function across them. Studies in the inter-connection of brain regions and corresponding molecular networks highly rely on bioinformatics. For instances, rank-rank hypergeometric overlap (RRHO) is a suitable approach that successfully revealed overlapping transcriptional patterns across brain regions (Bagot et al., 2016; Labonté et al., 2017; Peña et al., 2017). Weighted gene co-expression network analysis (WGCNA) is another widely used approach to explore gene networks involved in several CNS disorders, including Alzheimer’s disease and schizophrenia (Rangaraju et al., 2018; Radulescu et al., 2020).

In the present study, we used an MPTP-induced mouse model of PD to evaluate the effect of treadmill exercise on motor ability. Our results demonstrated a better performance in movement and balance, for the “MPTP + Exercise” group compared to the “MPTP” group. Then, we utilized RNA-sequencing to generate transcriptional profiles in the SN, striatum, cortex, and cerebellum for control, MPTP, and exercised PD model. RRHO and WGCNA were further applied to identify brain region-specific patterns and networks of co-regulated genes associated with exercise. Our data suggested an overlapping transcriptional pattern in the SN and striatum, and an opposite regulation in the SN and striatum after exercise. Pathways analysis revealed synaptic-related processes, especially synaptogenesis in the striatum was upregulated. Finally, we performed immunofluorescence experiments and validated that MPTP treatment reduces the number of striatal TH^+^ synapses while treadmill exercise restores it. Overall, this work provides a novel understanding in the molecular basis of PD exercise by leveraging a systematic and unbiased approach on transcriptional regulation, and may further promote the development of anti-PD strategy.

## Materials and Methods

### Animals

C57BL/6J mice (8-week-old, female) were purchased from Charles River, Beijing. Mice were maintained at 22 ± 1°C on a 12-h light/dark cycle. All experiments were conducted following international standards on animal welfare.

### Chronic Mouse Model of Parkinson’s Disease

After 1-week adaptation, mice were injected with MPTP (25 mg/kg, 10 doses, s.c.) in combination with probenecid (Dauer and Przedborski, 2003). During the chemical preparation and animal injections, safety precautions for MPTP use were observed (Jackson-Lewis and Przedborski, 2007).

### Treadmill Exercise Protocol

After the entire course of MPTP, the PD mice in the exercise groups (PD + EX) were forced to run on a treadmill (Duanshi, China) for 5 days/week, 40 min/day with a speed up to 15 m/min (5 min at 6 m/min, 5 min at 9 m/min, 20 min at 12 m/min, 5 min at 15 m/min, and 5 min at 12 m/min) for 12 consecutive weeks (Lau et al., 2011).

### Motor Behavior Analysis

After the entire course of MPTP and exercise, the mice were evaluated for their motor dysfunction by open field, rotarod, and pole test. In the open field, the mice were placed in a 100 _ 100 cm field individually for 15 min between 9 and 12 am (Huang et al., 2015). Then, we used the image processing system Xeye Aba 3.2 to analyze average velocity. In the rotarod test, mice were placed on a rotarod over 300 s at an accelerating speed (from 5 rpm to 50 rpm) (Petroske et al., 2001; Meredith and Kang, 2006). In the pole test, the time when mice were taken from the top of the pole for 3 times was recorded, then the average time was calculated (Ogawa et al., 1985; Matsuura et al., 1997).

### Brain Tissue Preparation

Following the final behavioral test, all the animals (Saline: *n* = 4, MPTP: *n* = 4, MPTP + EX: *n* = 4) were euthanized *via* cervical dislocation. the SN, striatum, cortex, and cerebellum samples were micro-dissected from the brain. Then, they were frozen at −80°C until the RNA extraction was performed. The cohort of animals (Saline: *n* = 5, MPTP: *n* = 5, MPTP + EX: *n* = 5) were anesthetized and then perfused transcranially with 4% paraformaldehyde and 0.1 M PBS. Then the brain was kept in 4% paraformaldehyde for at least 8 h, then cryoprotected in 20 and 30% sucrose for 48 h. Finally, brains were sliced into 15 μm sections for staining.

### RNA Isolation

Ice cold TRIzol Reagent (Life Technologies) was added to frozen tissue on ice. The total RNA was extracted following the manufacturer protocol by TRIzol Reagent. RNA concentrations were assessed by Nanodrop 2000/2000C, and RNA quality was checked by the Agilent 2100 Bioanalyzer.

### Transcriptome Analysis

After RNA-seq library preparation, all reads were mapped to *Mus musculus* as the reference genome by Tophat2 (v2.01). Then, we normalized FPKM (Fragments Per Kilobase of transcript per Million fragments mapped) of genes were estimated by Cuffquant (Cufflinks v2.21). Then, we calculated the number of FPKM by RSEM (V1.2.4) software. We used DESeq (V1.14.0) software to identify DEGs which FPKM > 1 and *p* < 0.05. We used the RRHO test to identify the degree of overlap in gene signatures among the brain regions in PD and exercised PD mice. WGCNA was used for the co-expressed gene modules. Pearson correlation coefficient analysis was performed for various groups and brain regions. Enrichment analysis, including GO analysis and KEGG pathway analysis, were performed by DAVID.

### Immunofluorescence

Six slices that extended through the regions of interest, including the cortex, cerebellum, SN, and striatum, were collected for TH^+^ immunofluorescence. All slices in the striatum were incubated in chicken anti-Tyrosine Hydroxylase (1:250, abcam, Cambridge, United Kingdom), rabbit anti-PSD95 (1:1,000; Synaptic Systems, Göttingen, Germany), and mouse anti-synapsin antibodies (1:1,000; Synaptic Systems, Göttingen, Germany) overnight at 4°C. After washing in PBS three times, sections simultaneously treated with 488-conjugated donkey anti-chicken IgG (1:1,000 dilution; Vector Laboratories Newark, CA, United States), 568-conjugated donkey anti-rabbit IgG (1:1,000 dilution; Vector Laboratories Newark, CA, United States), and 647-conjugated goat anti-mouse IgG (1:1,000 dilution; Vector Laboratories Newark, CA, United States) for another 1 h at room temperature. Then, the slices were observed under a confocal laser scanning microscope. Images were processed with software Imaris (Bitplane, Schlieren, Switzerland).

### Statistical Analysis

All data were expressed as mean ± SEM. Normal distributions and equal variances were determined for all data sets. A one-way or mixed-model ANOVA was performed as required. Tukey’s test and LSD were followed to conduct multiple comparisons, respectively. For all data, *p* < 0.05 was considered to be statistically significant. Data analysis was performed by SPSS 19.0, and graphs were plotted by GraphPad Prism 7.

## Results

### Treadmill Exercise Improves Motor Dysfunction in Parkinson’s Disease Model

**FIGURE 1:**
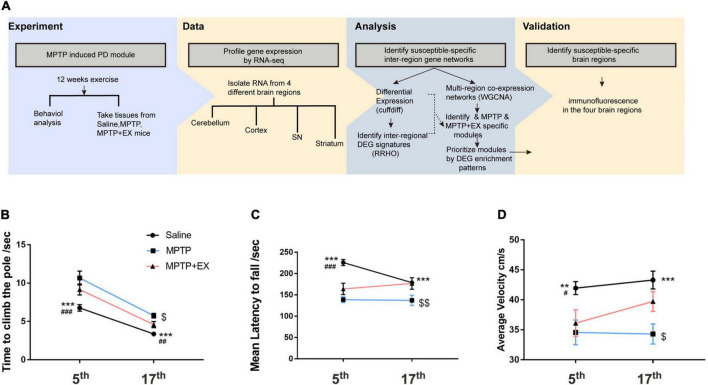
Motor function recovery in mice treated with MPTP after the 12-week treadmill exercise. **(A)** Scheme of the experimental design. **(B)** Time spent in descending to the floor for the saline (*n* = 24), MPTP (*n* = 12), and MPTP + EX (*n* = 12) groups. **(C)** Time spent in the rotarod for the saline (*n* = 24), MPTP (*n* = 12), and MPTP + EX (*n* = 12) groups. **(D)** Average speed in the open field for the saline (*n* = 24), MPTP (*n* = 13), and MPTP + EX (*n* = 13) groups. Data are mean ± s.e.m. Statistical analysis is performed using mixed-model ANOVA followed by the LSD *post hoc* test. **p < 0.01, saline vs. MPTP; ***P < 0.001, saline vs. MPTP; # *p* < 0.05, saline vs. MPTP + EX; ### *p* < 0.001, saline vs. MPTP + EX; $ *p* < 0.05, MPTP vs. MPTP + EX; $$ *p* < 0.01, MPTP vs. MPTP + EX.

After the 5-week MPTP treatment, we assessed the animals’ motor function by the rotarod test, the open field test, and the pole test (Figure 1A). Animals in the saline group exhibited significant different performance in behavior tests, compared to mice when MPTP treatment was just completed (Figures 1B–D). The two groups (MPTP and MPTP + EX) exhibited lower retention times on the rotarod test as compared to the saline group (Figure 1C). The pole test showed that both the MPTP and MPTP + EX group descend to the floor more slowly (Figure 1B) and exhibited a slower average velocity as compared to the saline group (Figure 1D).

After 12 weeks of exercise (treadmill training), we investigated whether that would alleviate motor deficits. Notably, the time to descend to the floor increased in the MPTP vs. Saline group (*p* < 0.001) and exercise drove a significant decrease compared to the MPTP group (Figure 1B, *p* < 0.05). The results unveiled a reduction in rotarod test time for the MPTP group compared to the saline group (Figure 1C, *p* < 0.001), and exercise reversed that tendency (Figure 1C, *p* < 0.01). In the open field test, average velocity was slowed in the MPTP vs. saline group (Figure 1D, *p* < 0.001), and exercise reversed that tendency (Figure 1D, *p* < 0.05).

### DEG Analysis Reveals Brain-Region-and Exercise-Specific Response Transcriptional Profiles in Parkinson’s Disease Mice

**FIGURE 2:**
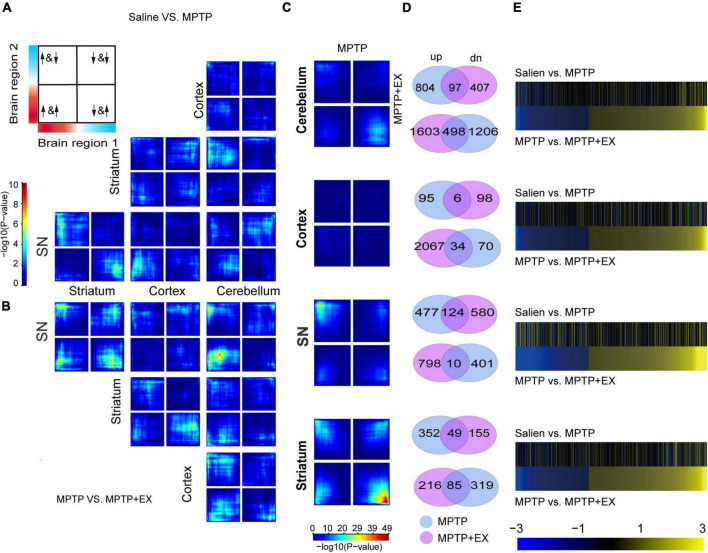
Inter-regional expression patterns reveals PD- and exercise-specific co-upregulation signatures. **(A,B)** RRHO maps comparing differential expression between 2 brain regions in PD mice [saline vs. MPTP, **(A)**] and exercised PD mice [MPTP vs. MPTP + EX, **(B)**]. The upper left in Panel A displays the overlap relationship across brain regions, and signals in the bottom left quarter show the genes overlap in common. The color bar spanning in Panel A represents *p*-values. **(C)** RRHO maps comparing differential expression between PD (saline vs. MPTP) and exercised PD mice (MPTP vs. MPTP + EX). **(D)** Venn diagrams display the overlap genes in MPTP and MPTP + EX in 4 brain regions. **(E)** Heat maps compare transcriptional changes [log(FC)] in PD to exercised PD mice across brain regions. For each brain region, the top pair of heat maps represents directionality of changes in PD mice for genes expressed in PD mice, while the bottom pair of heat maps represents directionality of changes for genes expressed in exercised PD mice.

To determine whether transcriptional profiles response to PD in a brain-region- and exercise-specific, we examined transcriptional profiles in 4 brain regions: cortex, cerebellum, SN, and striatum. For each condition and brain region, unbiased RRHO analysis was applied to reveal the transcriptional changes in 4 brain regions in MPTP mice. Results of RRHO analysis disclosed opposing up- and down-regulation of transcriptional signatures in the SN and striatum (Figure 2A), and a similar but weaker change for gene regulation in striatum and cerebellum (Figure 2A). Overlapping patterns were found across the cerebellum region. Our analysis revealed a coordinated up-and down-regulation of genes between the SN and striatum in MPTP + EX (Figure 2B). While the overlapping region between the cerebellum and SN was weaker in MPTP + EX (Figure 2B). Additionally, we detected minor overlap genes between MPTP and MPTP + EX transcriptional patterns in the cortex region, but stronger overlapping patterns in SN, especially in the striatum (Figure 2C). A comparison of DEGs in the MPTP vs. saline group and MPTP + EX vs. MPTP revealed overlapping across all 4 brain regions (Figure 2D). Moreover, the change directionality observed in the PD model and exercised PD model were different (Figure 2E). These analyses indicated that SN and striatum might have different responses to exercise in patients with PD.

### Remarkable Different Genes in SN and Striatum Regions Affected by Exercise for Parkinson’s Disease Mice

**FIGURE 3:**
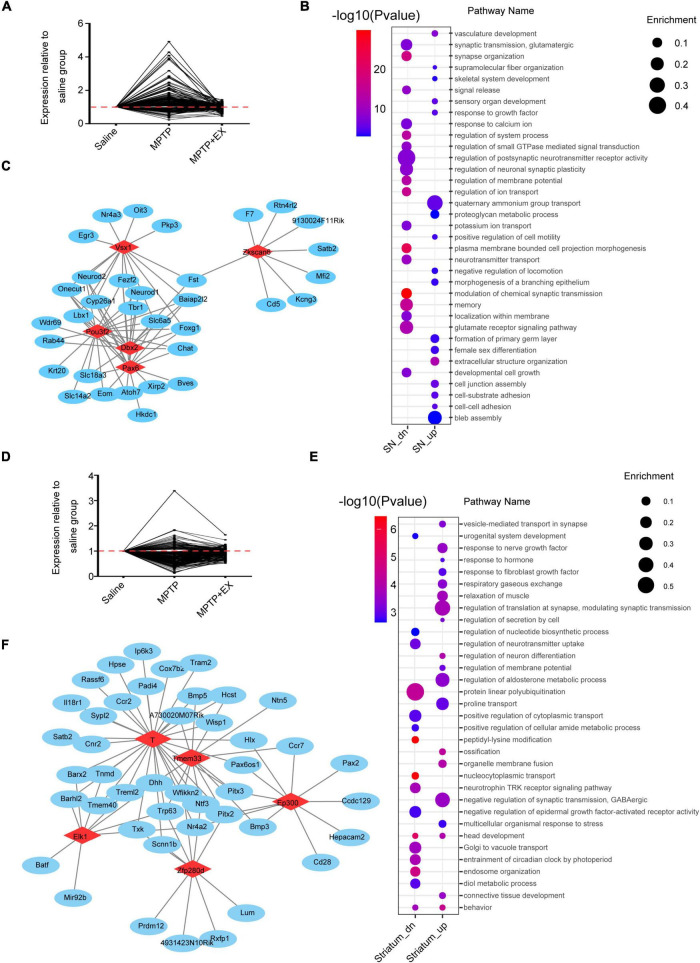
Remarkably different genes among the individual region. **(A)** Genes upregulated in the MPTP group (MPTP vs. saline) and downregulated in the MPTP + EX group (MPTP + EX vs. MPTP) in SN (FPKM > 1, *P* < 0.05). **(B)** Representative pathways enriched in SN (Enrichment: the ratio of observed gene numbers to expected gene numbers). **(C)** Transcription factor analysis in SN. Red nodes resemble transcriptional factors (TFs) and blue nodes are corresponding promoters, demonstrating a transcriptional regulation between TFs and genes. **(D)** Genes upregulated in the MPTP group (MPTP vs. saline) and downregulated in the MPTP + EX group (MPTP + EX vs. MPTP) in striatum (FPKM > 1, *P* < 0.05). **(E)** Representative pathways enriched in striatum. **(F)** Transcription factor analysis in striatum. Red nodes resemble TFs and blue nodes are corresponding promoters, demonstrating a transcriptional regulation between TFs and genes.

To further investigate the role of SN and striatum in PD and the exercise group for PD, genes with opposite expression patterns after modeling and locomotion were filtered. Genes selected in the SN are shown in Figure 3A. After exercise, 155 genes were down-regulated and 66 genes were up-regulated. Pathway analysis revealed several pathways enriched in PD model or exercised MPTP-induced PD model in SN. After exercise, the down-regulation of pathways were striking, including oxidative stress and apoptotic pathways. Meanwhile, we saw the up-regulation of memory, axon guidance, and synapse assembly pathways (Figure 3B). Furthermore, transcription factor analysis revealed that Pax6, Dbx2, Pou3f2, Zkscan6, Vsx1 were the top 5 remarkable transcription factors in SN. Their downstream genes were growth, development, and inflammation-related genes like Neurod2, Rtn4rl2, and Cd5, etc., (Figure 3C and Supplementary Table 2). In the striatum, 49 genes were down-regulated and 185 genes were up-regulated after exercise (Figure 3D). More pathways were enriched in up-regulated pattern after exercise. The enriched pathways were found to be related to neurogenesis, synaptogenesis, and transmitter transport like connective tissue development, head development, neuronal system regulation of neuron differentiation synapse organization, neurotransmitter receptor transport to the postsynaptic membrane (Figure 3E). Further analysis revealed that Ep300, Tmem33, T, Zfp280d, and Elk1 were the top 5 remarkable transcription factor binding sites in the striatum. Their downstream genes focus on development-related genes like Bmp5, Nr4a2, and Ntn5, etc., (Figure 3F and Supplementary Table 3). These results indicated that the microenvironment of DA neurons in SN might be improved, and synaptogenesis was promoted in the striatum after exercise in the PD model.

### WGCNA Identify Specific Transcriptional Signatures Associated With Exercise Rehabilitation in Parkinson’s Disease Model

**FIGURE 4:**
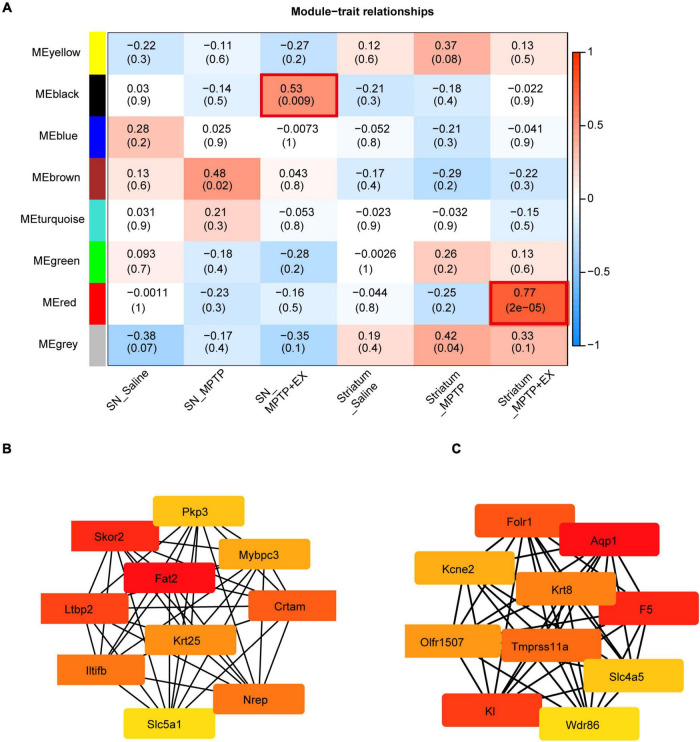
Identification of modules associated with the exercise treatment for PD mice in SN and striatum. **(A)** Heatmap of the correlation between the module eigen genes and exercise treatment of PD. We selected the MEblack-grade block and MEred-grade block for subsequent analysis according to the *p*-value and Pearson’s coefficient. **(B,C)** Hub genes analysis in the MEblack-grade block and MEred-grade block (ME: Module Eigen genes).

To further investigate transcriptional signatures in SN and striatum and gain more insights into the molecular mechanisms involved in PD’s exercise, we conducted WGCNA to detect gene co-expression network modules of saline, MPTP and MPTP + EX groups from multi-brain-region which combined SN and striatum. Our analysis identified gene co-expression modules, each name labeled by random color. As shown in Figure 4A, our analysis showed that the black module was positively correlated with exercise rehabilitation in SN, and MEred showed a positive correlation with exercise rehabilitation in the striatum (Figure 4A). Furthermore, our hub gene analysis exhibited the top 10 hub genes in the black model comprised of Fat, Skor2, Ltbp2, Crtam, Nrep, Lltifb, Krt25, Mybpc3, Pkp3, and Slc5a1. Most of these hub genes in this module encode cell adhesion and cell proliferation factors (Figure 4B). The Fat gene-encoded protein most likely functions, such as cell adhesion molecule, controlling cell proliferation, and playing an essential role in cerebellum development. The top 10 highly connected hub genes for the red model included K1, F5, Aqp1, Folr1, Tmprss11a, Krt8, Kcne2, Olfr1507 Slc4a5, and Wdr86, which were associated with synaptic vesicle transport (Figure 4C). These analyses further indicated improved synaptogenesis in the striatum after exercise in the PD model, which is deeply associated with behavior improvement.

### Exercise Increased TH^+^ Fibers and Synapses Obviously in the Striatal Region but Failed to Increase DA Neurons in the SN of Parkinson’s Disease Model

**FIGURE 5:**
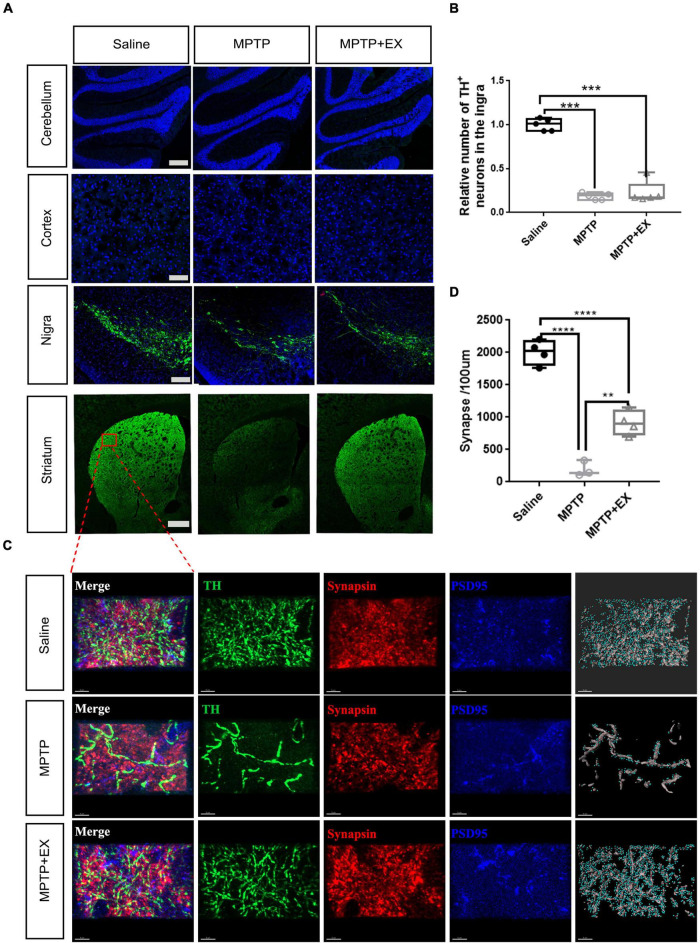
Immunohistochemical staining experiments validate synaptogenesis involved in exercise-induced PD recovery. **(A)** TH^+^ staining in the cerebellum (scale bar, 200 μm), cortex (scale bar, 200 μm), SN (scale bar, 200 μm), and striatum (scale bar, 500 μm). **(B)** Relative number of TH^+^ cells in the SN for Saline, MPTP, and MPTP + EX groups (*n* = 5 per group). **(C)** Immunohistochemical staining of TH^+^ fibers (green) stained with synaptic markers in striatum (scale bar, 5 μm). Presynaptic terminals and postsynaptic structures were stained by synapsin (Syn, red) and postsynaptic density protein 95 (PSD95, blue), respectively. Synapses were identified by the close proximity of pre- and postsynaptic elements (< 1 μm). A 3D construction of PSD-95 and presynaptic synapsin was performed with “create spots” algorithm in Imaris (scale bar, 5 μm). **(D)** Quantitation of synapses in the striatum for saline, MPTP, and MPTP + EX groups (*n* = 3–5 per group). Data are mean ± s.e.m. Statistical analysis was performed using one-way ANOVA followed by Tukey’s *post hoc* test; **p < 0.01, ***p < 0.001, ****p < 0.0001.

The previous results demonstrated an improvement in locomotor performance after exercise intervention in the PD model. Our results showed up-regulation of synapse-related genes and pathways in the striatal region after the motor intervention and an improvement in the microenvironment in SN. These suggested an improved state of DA neurons and increased number of related synapses. To further test our hypothesis, we performed TH staining in four brain regions. As seen in Figures 5A,B, immunofluorescence processing of SN sections showed a 70% decrease of TH positive neurons after MPTP administration in sedentary and exercise animals compared to the saline group (*P* < 0.001). Results of immunofluorescence also showed that while there was a hint of recovery of TH^+^ cells in the SN after exercise, it was not statistically significant (Figure 5B). Fluorescence intensity in striatal areas showed an enhancement after the exercise intervention, but no corresponding change was detected in the motor cortex or cerebellum (Figure 5A). To further test our hypothesis that the increased fluorescence intensity in the striatal region resulted from the improved synaptic condition of DA nerve fibers, we performed TH^+^ fiber (green) and synaptic marker staining in the dorsolateral striatal. The staining and statistical results showed that the synapses recovered to half of the control group after the exercise intervention (Figures 5C,D).

## Discussion

In this study, molecular network models were developed to extend the understanding of transcriptional mechanisms of exercise for PD. The whole transcriptome in 4 inter-connected brain regions related to PD and locomotor activity was detected by performing a co-expression network analysis. Initial results suggested the crucial role of SN and striatum in exercise for PD mice, rather than cortex and cerebellum. Pathways analysis reveals up-regulation in synapse-related pathways in SN and up-regulation of synaptogenesis in the striatum, which was further confirmed by immunofluorescence in striatum.

Previous clinical and animal studies of mechanisms in PD and exercised PD mice have focused on individual candidate genes, or one single brain region in isolation. For example, MRI observed that the caudate nucleus and putamen in the striatum were significantly atrophic and PD (Barbagallo et al., 2016). Impaired higher cortical function was found in patients with PD and FMRI showed enhanced connectivity in the M1 region of the motor cortex after an 8-week exercise treatment in patients (Messa et al., 2019). Moreover, decreased dopamine receptors D1 and D3 in the cerebellum was found in patients with PD (Yang et al., 2021). RRHO and WGCNA have been previously applied to generate insight into other diseases. Although those observations disclosed some key molecules involved in the PD pathophysiology, they did not exhibit enough evidence in circuitry dysfunction, which was also considered a critical characteristic for subjects with PD (Petersson et al., 2019, 2020). Here, we successfully identify novel transcriptional networks associated with exercised PD mice by RRHO and WGCNA. We found a similarity of transcriptional regulation between SN and striatum in PD with the exercise group. The interaction between those two brain regions probably arose from the nigrostriatal pathway, in which the loss of dopaminergic neurons was considered as the hallmark of PD. For example, acute α-synuclein preformed fibrils (PFFs) spreading along nigrostriatal path impaired neurotransmission and microglial reactivity in adult mice (Sun et al., 2022). Lahiri and Bevan presented direct evidence that stimulating nigrostriatal dopamine axons persistently enhances the intrinsic excitability of direct pathway striatal neurons (Lahiri and Bevan, 2020). Moreover, several studies implied that physical exercises reduce motor deficits in Parkinson’s disease through rescuing lesions in nigrostriatal dopamine (DA) system (Toy et al., 2014; Hou et al., 2017; Shi et al., 2017). Our results are consistent with those observations, which suggest a fundamental role of nigrostriatal pathway in regulating motor symptoms in PD.

Results from pathway analysis showed that, in the SN, exercise suppress oxidative stress and apoptotic pathways, whereas it upregulates memory, axon guidance, and synapse assembly pathways, which promote neuronal growth, development, and inhibit inflammation (Hirsch and Hunot, 2009). In the striatum, neurotrophic factor response pathways, neurogenesis, synaptogenesis, and transmitter transport-related pathway were up-regulated after exercise. The enrichment of those molecules would improve the microenvironment of the SN and facilitate the synapse formation in the striatum, according to previously published literature (Petzinger et al., 2013; Mak et al., 2017). WGCNA analysis further conformed to the association between exercised phenotype and pathways, and further screened for critical modules and hub genes. Some of hub genes shown in Figures 4B,C have been reported in previous studies of PD: Krt25 is implicated in motor coordination (Zhang et al., 2018); Aqp1, encoding astrocytic water channel proteins aquaporin 1 (AQP1) might profoundly affect α-syn deposition in the neocortex of patients with PD (Hoshi et al., 2017); FolR1 is selectively expressed in the surface of midbrain DA progenitors and its level may represent the condition of TH positive neurons (Gennet et al., 2016). Comprehensively considering their functions, we proposed that the hub genes manipulate behavioral susceptibility by driving synaptogenesis, neuronal network formation, and improving microenvironment in the nigrostriatal system after exercise. From the results, we suspected that the generation of compensatory neuron adaptations is most likely to occur by exercise for PD mice.

In immunofluorescence assay, we found exercise restored the synaptic density in the striatum of MPTP-treated animals, although the loss of TH positive neurons was not affected (Figure 5). This observation not only validated the key molecular pathways obtained from WGCNA, but also demonstrated a reconstruction of neural circuits in striatum during the physical exercise. Recent studies have identified axonal dysfunction in the SN as a typical feature for PD (Sanchez-Catasus et al., 2021; Wang et al., 2022). The synapse regeneration in striatum after exercise in the present study also represents the recovery of nigrostriatal pathway. Nevertheless, the underlying mechanism of reconstructing nigrostriatal neural circuits after exercise has not been known yet. Although inhibition of SN microglia activation has been proposed to play a role (Wang et al., 2022), further investigations are still needed to achieve comprehensive conclusions and to develop effective anti-PD strategy.

In the present study, mice were treated with high-intensity aerobic training; thus the outcome of exercise intervention in patients with PD may also be deeply associated with practice intensity. In addition, changes in those four brain regions would not cover all exercise-induced effects for PD. Other regions, such as pedunculopontine nucleus (PPN), controlling the gait initiation, modulation, and other stereotyped movements might be also involved. Previous animal studies have shown the degeneration of PPN neurons may trigger the impairment of locomotor and postural disturbances in PD (Gai et al., 1991; Masini and Kiehn, 2022), and “bursting” glutamatergic PPNd neurons are related to the initiation of programmed movements while non-bursting cholinergic PPNc neurons are related to the maintenance of steady-state locomotion (French and Muthusamy, 2018). As gait performance and mobility of PD patients can be improved after physical training (Mak et al., 2017). Therefore, PPN may act as another target region of the brain for exercise-induced rehabilitation in PD, and studies upon molecular mechanisms in PPN are promising. Last but not least, cell-type-specific alterations in transcriptome profile should also play a role in PD progress or rehabilitation (Smajiæ et al., 2021), but those were not investigated in this research. Further studies utilizing single cell RNA sequencing in brains can be performed to further elucidate the mechanism in PD rehabilitation after physical exercise.

## Conclusion

This study provides a powerful example of four brain regions in PD and exercised PD mice by integrating large-scale transcriptomic data. We provided evidence that exercise contributes to the recovery of motor deficits in PD, probably *via* modulating connections between the SN and striatum. Pathways in neurogenesis, synaptogenesis, transmitter transport, axon guidance, and synapse assembly are critical for exercise-induced benefits.

## Data Availability Statement

The datasets generated for this study can be found in Gene Expression Omnibus (GEO), GSE205907.

## Ethics Statement

The animal study was reviewed and approved by Tongji Hospital, School of Medicine, Tongji University, Shanghai, China.

## Author Contributions

WT and HY made the PD mouse model. WT, YH, and QG trained the mice. WT and KZ analyzed the RNA-sequence data. WT and LL performed behavioral tests. WT, JZ, and YS conducted immunofluorescence. YES, SL, and LJ designed the experiments and wrote the manuscript. All authors contributed to the article and approved the submitted version.

## Conflict of Interest

The authors declare that the research was conducted in the absence of any commercial or financial relationships that could be construed as a potential conflict of interest.

## Publisher’s Note

All claims expressed in this article are solely those of the authors and do not necessarily represent those of their affiliated organizations, or those of the publisher, the editors and the reviewers. Any product that may be evaluated in this article, or claim that may be made by its manufacturer, is not guaranteed or endorsed by the publisher.

## Supplementary Material

Supplementary Figure 1 Heatmaps of differentially expressed genes (DEGs) in cerebellum **(A)**, cortex **(B)**, SN **(C)**, and striatum **(D)**. The color bar represents log_2_ (FPKM).
