# Supplementary figures and images for: Transcriptional Profiling Reveals Brain Region-Specific Gene Networks Regulated in Exercise in a Mouse Model of Parkinson’s Disease
Source: Front Aging Neurosci. 2022 Jun 23;14:891644. doi: 10.3389/fnagi.2022.891644 (PMC9260255; doi:10.3389/fnagi.2022.891644)

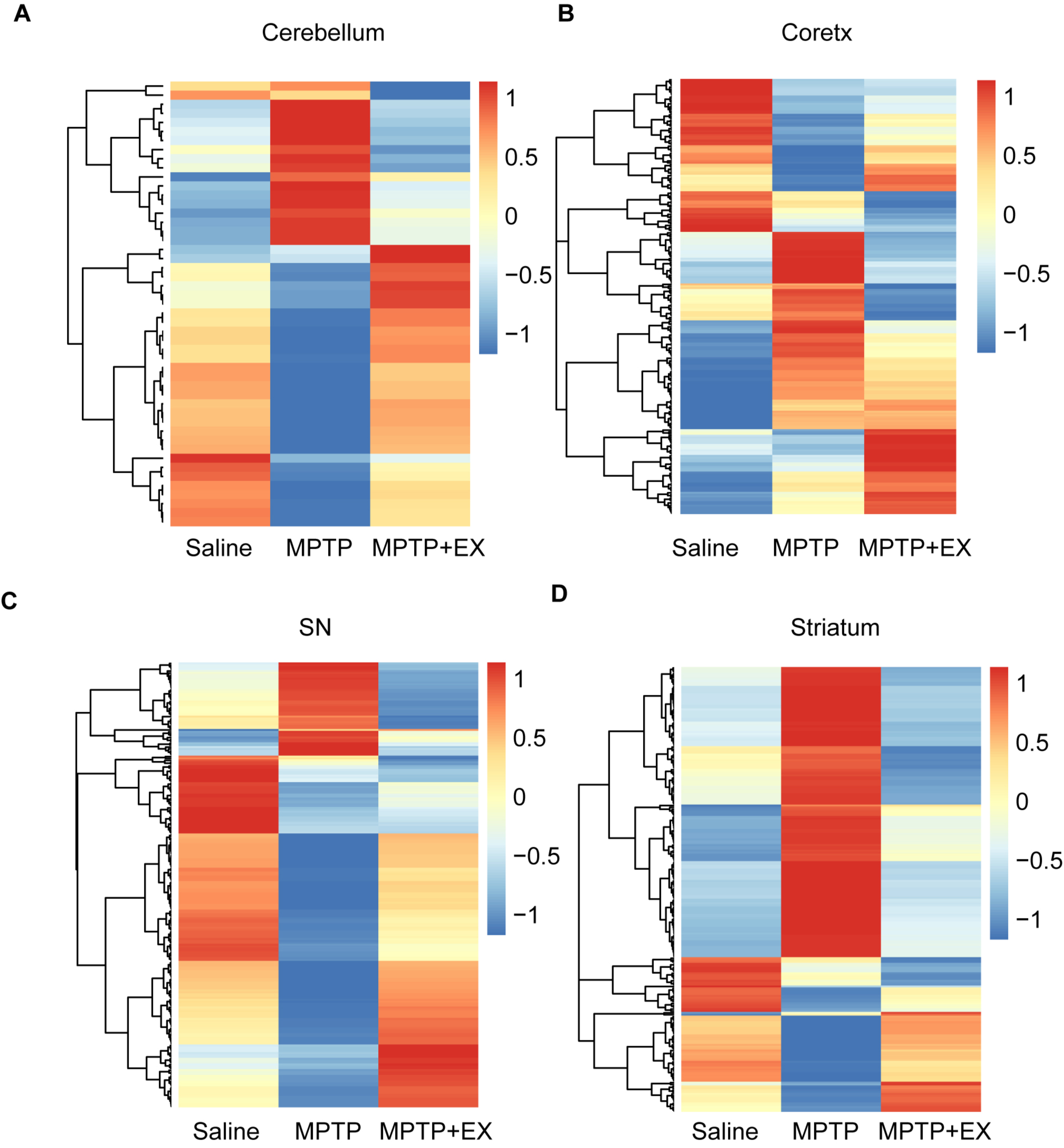

Supplement: Supplementary Figure 1 — Heatmaps of differentially expressed genes (DEGs) in cerebellum (A), cortex (B), SN (C), and striatum (D). The color bar represents log2 (FPKM). [file Image_1.jpg]
